# News Article Portrayal of Virtual Care for Health Care Delivery in the First 7 Months of the COVID-19 Pandemic

**DOI:** 10.1089/tmr.2020.0033

**Published:** 2021-03-23

**Authors:** Kathy L. Rush, Lindsay Burton, Mindy A. Smith, Sarah Singh, Kira Schaab, Matthias Görges, Robert Janke, Leanne M. Currie

**Affiliations:** ^1^ School of Nursing, University of British Columbia-Okanagan, Kelowna, British Columbia, Canada.; ^2^ Patient Voices Network, Kootenay-Boundary Collaborative Services Committee, Vancouver, British Columbia, Canada.; ^3^ Department of Family Medicine, Michigan State University, East Lansing, Michigan, USA.; ^4^ University of Medicine and Health Sciences, Basseterre, Saint Kitts and Nevis.; ^5^ Department of Anesthesiology Pharmacology and Therapeutics, University of British Columbia, Vancouver, British Columbia, Canada.; ^6^ Research Institute, BC Children's Hospital, Vancouver, British Columbia, Canada.; ^7^ Library, University of British Columbia-Okanagan, Kelowna, British Columbia, Canada.; ^8^ School of Nursing, University of British Columbia, Vancouver, British Columbia, Canada.

**Keywords:** virtual care, newspaper, qualitative analysis

## Abstract

**Objectives:**

The onset of the 2020 coronavirus pandemic resulted in rapid implementation of virtual care solutions at an unprecedented pace. The news media, as a trusted source for many Canadians, plays a vital role during emergencies by reporting on changes in health care protocols, policies, and technologies. This article presents the results of a qualitative analysis of Canadian news articles between February and August of 2020 to identify critical themes with respect to virtual care.

**Methods:**

A full-text search of the database Canadian Newsstream resulted in 1542 articles (708 duplicates), of which 294 articles were included in the final analysis. Inductive analysis was used to generate themes and identify voices, contradictions, and tensions in the articles.

**Results:**

Analysis generated four themes: coronavirus disease (COVID-19) as a catalyst for virtual care, safety and protection, economic impacts, and telehealth as a model of care. Media portrayals represented some voices (e.g., physicians) while limiting others (e.g., patients), reflected some contradictory messaging with respect to safety and protection, and raised key issues and concerns about virtual health care delivery during the first 7 months of COVID-19.

**Conclusions:**

Our findings of successful and rapid uptake, uses and concerns around funding, and privacy and virtual care adoption reported in the news media can be used to inform longer term implementation and sustainability. Policy makers could benefit from crafting messages that balance information and reassurance. Public/patient perspectives, which were largely missing from news media, are needed to gauge receptivity and sustainability.

## Introduction

The media plays an indispensable role during emergency response as reporters interpret, collate, process, and transmit news using both positive and negative messages.^1^ With the rapid evolution of the coronavirus disease (COVID-19) pandemic, there has been a mass turnover of news reporting on changes in policies, protocols, technologies, and health services.

One significant change has been the explosive uptake of virtual care to comply with social distancing recommendations and respond to changes in safety protocols while ensuring ongoing patient care. Virtual care is broadly defined as use of technology to support health care activities not carried out in-person, including videoconferencing (telemedicine, telehealth), telephone (telehomecare, nurse call centers), e-mail, and text messaging.^2^ The pandemic is likely a game-changer in setting standards for future health care delivery.^3^ Understanding messaging during this critical time provides insight into the adopted features, expectations, and value placed on virtual care.

Media sources have a potent influence on public opinion, policy, and practice through selective communication about certain aspects of a perceived reality that become salient in the thinking and views of the public.^4^ During the pandemic, social media has been examined for lay perspectives and regulations,^5^ and medical professional opinions and recommendations.^6^ News media has received less attention but also contributes importantly to public perception.

News media is a trusted information source for Canadians, and most (88%) Canadians read the paper and digital newspapers weekly.^7^ In addition, newspapers attract important demographics; a report from the Statista Research Department found that the highest newspaper readership across Canadians belonged to consumers between the ages of 24–49 years (23.89%), followed by those older than 64 years.^8^ In contrast, news readership on social media sites such as Reddit, Facebook, and Twitter, while attracting those aged 30–49 years (38–40% of readership), attracts a younger demographic (26–52%) and has few readers older than 64 years (2–11%).^9^ Interestingly, most readers of news on social media in both the United States and Canada do not view these platforms as trustworthy news sources in contrast to 72% of Canadians trusting local or regional newspaper reporting.^9,10^

To our knowledge, there has been no study of media coverage related to virtual care in Canada or elsewhere. The purpose of this study was to identify critical themes regarding virtual care during the first 7 months of the COVID-19 pandemic communicated in Canadian news articles and to explore different perspectives and barriers and facilitators that might influence future use.

## Methods

### Design/sampling/data extraction

A qualitative approach was used to guide the study. We searched Canadian Newsstream, a comprehensive database containing over 360 news sources published in Canada, inclusive of small-town local newspapers to large national newspapers. For the purpose of this article, we defined news media as print and online indexed newspaper articles published in Canadian newspapers. Although Canadian Newsstream contains archives of newspapers back to the 1970s, it is also updated daily making its breadth, depth, and currency of searchable new sources unparalleled. Because of the focus of this investigation, only articles from February 1, 2020, to August 31, 2020, were included.

The search strategy, developed in consultation with a research librarian and subject matter experts on the team, included the following terms: (telemedicine or telehealth or “virtual medicine” or ehealth or mhealth or “virtual care” or Teledoc or “health technology”) AND (COVID or “COVID-19” or coronavirus or pandemic). Duplicates were removed and articles were screened for relevance. Inclusion criteria were as follows: newspaper articles, reference to virtual care, Canadian context, and English language. Exclusion criteria were as follows: classified advertisements and articles only reporting COVID-19 cases. Data were extracted and imported to NVivo 12 for analysis. Three research members (S.S., K.S., and L.B.) extracted information on province, publication date, and perspective, as shown in Table 1.

**Table 1.** Descriptive Summary of News Articles

Characteristic	*n* (%)
Location of publication	
Alberta	36 (12.2)
British Columbia	46 (15.6)
Manitoba	7 (2.4)
New Brunswick	20 (6.8)
Newfoundland and Labrador	—
Northwest Territories	—
Nova Scotia	11 (3.7)
Nunavut	—
Ontario	155 (52.7)
Prince Edward Island	—
Quebec	6 (2.0)
Saskatchewan	9 (3.1)
Yukon	4 (1.4)
Month published	
February	2 (1.4)
March	66 (22.4)
April	68 (22.6)
May	78 (26.5)
June	26 (8.8)
July	36 (12.2)
August	19 (6.5)
Perspective	
Reporter	244 (83.0)
Physician	128 (43.5)
Health executive	74 (25.2)
Politician	39 (13.3)
Health authority	35 (11.9)
Provider (general)	35 (11.9)
Tech company	29 (9.9)
Patient	23 (7.8)
Clinic	12 (4.1)
Other	43 (14.6)

### Data analysis

We conducted inductive analysis using Altheide and Schneider's qualitative media analysis guidelines.^11^ Analysis also attended to who spoke, how they spoke (defined as overall article tone), and to whom or about whom they spoke; whose voices were not heard or not (were there quotes from or interviews directly with patients, providers, organizations, or company representatives); and the contrasts, contradictions, and tensions in what was being said. While more subjective, we characterized article tone as positive if the report was portraying a hopeful, excited, or positive messaging; negative if the report was critical, commanding, or explaining disappointment or concerns; or neutral if nonopinionated, which were primarily in instructive or educational articles.

The same team members initially coded 10% of the articles for units of meaning (e.g., words, phrases, paragraphs) and clustered similar codes into themes and subthemes for coding the remaining articles. Coding was iterative with refinements made to account for the data and consensus on the final themes and subthemes achieved through ongoing team discussion. Based on a random selection of 20% of news articles, calculated Cohen's Kappa (k),^12^ produced high intercoder agreement (0.835–1.0).

## Results

Search results revealed 1821 articles; with duplicates removed (*n* = 708) and those that did not meet the inclusion criteria (*n* = 414), 294 articles remained. The largest volume of virtual health news was found in Ontario (ON), Alberta (AB), and British Columbia (BC), with no news presence appearing in Newfoundland, Prince Edward Island, and the Northwest Territories (Table 1). Article volume was highest from March 2020 through May 2020 and decreased substantially from June through August. Four main themes emerged: (1) COVID-19 as a catalyst for virtual care, (2) safety and protection, (3) economic impacts, and (4) virtual care as a care model.

### COVID-19 as a catalyst for virtual care

This theme, present in 24% of articles, described how COVID-19 removed virtual care barriers, or expanded and/or escalated virtual care use, enterprise, technology growth, or policy development. COVID-19 as a catalyst first appeared in March (*n* = 11), steadily increased until May (*n* = 20), and decreased during the summer months (*n* = 26). Emphasis was on clinicians' rapid uptake of virtual care, with physicians' perspectives dominating. Other unnamed clinical providers (e.g., physiotherapists, nurse practitioners) were mentioned secondarily.

This pan-Canadian catalytic growth was observed in provincial virtual care initiatives (e.g., Babylon, Telehealth Ontario), local community clinics, and in businesses/companies to meet demands. Growth was captured in nonspecific, superlative language such as increased use “as much as possible” and in numerical data showing exponential increases in virtual care use (Table 2).

**Table 2.** Catalyst for Care Theme with Supporting Quotes

Theme	Quotes
Catalyst for telehealth	*Physician early adopter*, “I knew patients would be on board. Doctors were another story, but COVID-19 kind of catalyzed it for physicians to see the value and to realize it's not as bad as they thought.” (The Toronto Sun, April 28)
	*Technology company CEOs*, “For various reasons we haven't had as much traction as we would have liked from the regulators and provincial health services. I think now there's a huge realization virtual health care has a huge role to play.” (The Globe and Mail, March 19); “Because of the crisis, normally slow-moving hospitals and health-care systems are ready to implement changes fast, and accept imperfections along the way.” (National Post, April 2); “(COVID-19) has been a major catalyst for the growth of virtual care. I believe it has fundamentally changed the adoption curve for digital services.” (The Province, April 5)
	*Reporter* (Virtual mental-health clinic): “An urgent care clinic like this had been proposed before so when the unprecedented need arose, Royal staff ‘blew the dust’ off that plan and forged ahead.” (The Ottawa Sun, April 6)
Meeting demand and numerical reports	*Reporters*, “Telehealth companies such as Montreal's Dialogue and Toronto-based MindBeacon are meeting surging demand from employers that want to provide remote care to housebound workers.” (The Globe and Mail, April 29). “One company providing a virtual care smart phone app, noted a doubling of demand for its services on ‘each of the past three days.’ Another company (TELUS Babylon) reported having triple the number of smartphone app downloads in one week at the beginning of the pandemic.” (The Globe and Mail, March 19).
	*Reporter,* “All scheduled appointments for the next two weeks are being converted to telemedicine consultations.” (Montreal Gazette, March 25).
	*Ministry of Health,* In January, Telehealth averaged about 1,900 calls a day, while in February it had about 1,600 daily calls. After the first 13 days in March, Telehealth is averaging 2,800 calls a day and “total call volume continues to see an upward trend” (The Globe and Mail, March 19; Toronto Star, March 15).
	*Executive Director of Shoreline Medical Society*, “40,261 telephone clinic appointments between March and August.” (Peninsula News Review, August 28)
Policy change	*Reporter*, “There are many exciting possibilities and good arguments for moving ahead on virtual-care protocols. But there are none better than the coronavirus outbreak, which has already prompted the system to work out a makeshift approach.” (Daily Gleaner, March 18)

Policy changes made by governments in BC, ON, New Brunswick (NB), and Nova Scotia (NS) emphasized the speed with which new policies were implemented to improve virtual care access. Policy and protocol changes were referred to in general terms, but all described COVID-19 advancing the virtual care agenda (Table 2).

### Safety and protection

This theme (48% of articles) encompassed the use of virtual care to comply with new guidelines to protect: (1) patient safety (80%), (2) provider safety (20%), and (3) the health care system (20%).^*^ Also noted were concerns about patient harm from potential confidentiality breaches related to virtual care. The volume of articles discussing safety and protection decreased by 43%, from 46 in March–May to 26 in June–August.

#### Patient focus

Virtual care use for patient safety was a primary emphasis. One patient described the benefits of not having to “get through several doors, touch many surfaces” (The Spectator, April 6). Public/patient safety and protection messaging regarding virtual care contained both instructional and reassuring communication (77 articles), and at times appeared conflicting (Table 3). For example, patients were instructed to stay away from clinics and hospitals while simultaneously being encouraged to seek care.

**Table 3.** Safety and Protection Theme with Supporting Quotes

Theme	Quotes
Patient safety	*Quebec physician*, “It's working very well. We're sparing patients from having to go outside and be exposed to people on the métro and on the bus. We're sparing people in the waiting room from picking up (the coronavirus).” (Montreal Gazette, March 25)
Instruction	*South Okanagan Similkameen Division of Family Practice in BC*, “A doctor or nurse practitioner will determine whether you can be seen over the phone, by video, or if an in-person appointment is required.” (Similkameen Spotlight, April 7).
Reassure	*Clinician*, “I would add that Crisis Text Line is very easy to use and could be a valuable first stop for someone in crisis. Simply text 741–741 and a volunteer social worker and crisis counsellor will respond very quickly via text. This service is free and available 24/7” (Sudbury Star, April 24)
	*Executive Director Kawartha North Family Health Team*, “In an effort to ensure everyone's safety, we ask that patients not come to the office without a pre-booked appointment.” (Kawartha Lakes This Week, May 4).
	*Chief of Staff*, “With all the focus on COVID-19, it can be easy for people's ongoing, regular health care concerns to be relegated to the back burner. I would like to assure you that you continue to have access to health care…” (100 Mile House Free Press, March 30)
	*Speech pathologist*, “…video therapy is as effective as in-person visits.” (Sault Star, April 1)
Provider safety	*Reporter*, “Virtual care can also help mitigate the risks posed to front-line health-care workers during a time of crisis. For those providers who are well but subject to quarantine, virtual care allows them to still contribute to health-care delivery through eConsult, video visits, email or chat communication, and remote monitoring, as long as appropriate cybersecurity measures are in place.” (Toronto Star, March 18)
	*Emergency department nurse*, “I'm not exactly scared. Not yet. I have anxiety, but I'm not necessarily afraid. The anxiety centers on the lack of available protective gear.” (Globe & Mail: April 4, 2020)
	*Presidents, Edmonton Zone Medical Staff Association and Edmonton Community Medical Staff Association*, “Family physicians have not received clinic guidelines nor a plan from the government for how the health system and family clinics will prepare to manage the additional volume. Family clinics do not receive [PPE] from government while hospitals and COVID-19 assessment centres do.” (Edmonton Journal, August 29)
Health care system	*Clinic spokesperson*, “Our goal is to minimize potential exposure to as many people as possible while freeing up resources to attend to those who require urgent care.” (The Standard, April 2).
	*Physician and President of the NB Medical Society*, “We are urging all our members to look at their individual practices and continue to practice as much social isolation as possible. It's hopefully going to reduce greatly the number of patients coming to physicians' offices and coming to hospitals.” (The Times – Transcript, March 17)
	*Physician*, ““…a system called Virtual Consults, which allows specialists to assess and treat a patient in the emergency department by video chat when an in-person assessment isn't necessary. This saves PPE, reduces exposure for everyone as fewer specialists are in the department and gives families a plan more quickly than if they had to wait for someone to come in.” (The Globe and Mail, July 18)

##### Instructional

Instructional messaging aimed to reduce COVID-19 exposure. Communications (e.g., lists, quotes, letters from experts) gave the public/patients directions for booking appointments, receiving care, accessing screening tools, and seeking medical advice. Of the 53 articles containing instructional messaging, the majority were published in March (*n* = 15), April (*n* = 14), and May (*n* = 16) and decreased substantially in June (*n* = 3), July (*n* = 2), and August (*n* = 3).

Over half of instructional messaging came primarily from identified physicians and health authorities, with 24 articles in which sources were not identified. Often, instructional messaging appeared at the beginning or end of articles, to capture attention. These messages used emphatic and commanding tones such as clear language that patients should not “drop-in” or go to offices without calling ahead (Table 3).

##### Reassurance

Reassurance messaging framed virtual care as a means for patients to continue receiving care while minimizing risk. Often appearing at the end of articles, reassuring messages were predominantly physician-relayed, encouraging patients to reach out for needed care, that virtual care was available, and that their usual needs were important despite the pandemic (Table 3).

Of the 37 articles containing reassuring messaging, none was published in February, five were published in March, 13 in both April and May, three both in June and July, and none in August. Although in-person care was available during the pandemic, the need for triaging (determining if the visit should be virtual or in-person) was also stated. Messages also reassured the public about the ease of access to non-in-person help, such as crisis lines.

#### Provider focus

Fewer articles referred to provider safety and protection conferred by virtual care. Articles described a greatly reduced number of in-person appointments and increased virtual care appointments to protect health care and frontline workers. Articles also gave voice to provider-expressed concerns regarding contracting COVID-19 and demand/supply issues for personal protective equipment (PPE) (Table 3); six of the seven articles with this content appeared after April 2020.

#### Health care system

Articles highlighted a virtual care role in protecting the health care system from being overwhelmed and “flattening the curve,” decreasing emergency department (ED) and physician-office traffic, and reserving these spaces for those in most need. Articles also described how moving to virtual care helped reduce burden on resources such as PPE and lowered transmission risks (Table 3).

### Economic considerations

The pandemic's economic impact (37% of articles) was considered from the lens of government (67%), provider (38%), and patient (15%). The majority of these articles were published between March and April, as funding decisions were being made, with a 30% decrease in the number of articles between June and August.

#### Government funding

Provincial government responses to virtual health care during the pandemic varied regarding the timing and type of investment. In Saskatchewan (SK), the government invested early, adding “$3.6 million for improving eHealth infrastructure.” (Star—Phoenix, March 19). In other provincial news, references appeared regarding the government's legislative, indirect support of virtual care in BC, SK, ON, Quebec (QC), NB, and NS. In BC, news articles described the government working with private medical insurance companies to ensure coverage for virtual allied-health services such as counseling and physiotherapy. The majority of articles covering government funding were published in March.

Government investment in ON, AB, and NS was not immediate. After introducing a new virtual care legislation (March 14, 2020), ON increased “…public funding by $160 million to support COVID-19 monitoring and testing, including investments in virtual care and Telehealth Ontario.” (Caledon Enterprise, March 31).

The AB government's pre-COVID-19 investment of $1.5 million/year in TELUS's Babylon app supporting provider-based virtual care (not the patient's own physician) delayed COVID-19-related investment in virtual care (Table 4). Many articles included interviews with AB physicians who were irate, claiming this app to be a “…push for privatization…” (The South Peace News, March 25) or a chance to enrich corporate shareholders. While provider comments reported in mid-March were mostly negative, comments were positive once government investments were announced (Table 4).

**Table 4.** Economic Considerations and Supporting Quotes

Theme	Quotes
Government support	*Edmonton physician*, “I'm concerned that they could probably have utilized that money going to TELUS and used it to a better fashion in our present clinics to manage patients (virtually).” (The Edmonton Sun, March 21).
	*President, Doctors of BC*, “…We're pleased that government has agreed to expand virtual care fees to include telephone services as well as suspend the daily volume limit. This includes consultations, office visits, and non-procedural interventions - for any health concern patients are wanting to see their doctor for.” (The Globe and Mail, March 17)
Provider	*Ontario physician*, “…he should be able to give advice and adjust prescriptions by telephone, rather than make these vulnerable patients come into his office for a face-to-face meeting.” (Waterloo Region Record, March 12)
	*Ontario physician* “…(delayed compensation for telehealth) dissuades people from this model. [It] is really mixed messaging.” (The Toronto Sun, April 28)
	*Nova Scotia psychiatrist*, “This (excluding mental health services) discriminates against patients with mental health problems and the psychiatrists who treat them.” (Chronicle—Herald, March 23)
	*Alberta physician*, “If a doctor completes 40 calls in one day, this would be $800, which is not enough to sustain a practice with staff, rent and bills.” (The Edmonton Sun, March 17)
	*Asclepios Medical Centre in Ontario*, “The closure is the result of the clinic simply not having the resources to sustain the increased operational costs and decreased revenues caused by the COVID-19 pandemic.” (The Ottawa Citizen, June 1)
	*Alberta physician*, “I am very grateful that the Alberta government has made these changes and is willing to work with the Alberta Medical Association.” (The Medicine Hat News, March 25).
Patient	*Private insurer*, “Now everyone with plans administered through HBT [Health Benefits Trust] will have these virtual care appointments - but only for preexisting conditions. PBC [Pacific Blue Cross] will only cover subsequent visits, not new injuries.” (The News, April 2)
	*Physiotherapist*, “…she had run into situations where a client's insurance covers the appointment, but they can't afford to make the payment and get reimbursed later.” (Kings County Record, April 28)

#### Provider economics

Provider virtual care compensation emerged as the most highly charged news item in the first few months of the pandemic due to highly variable provincial changes in billing codes and legislation. Articles from BC, SK, and QC noted immediate changes (mid-March) to physician compensation for virtual care services.

In contrast, AB, ON, and NS reported delays in virtual care provider compensation and limited compensation for mental health services, resulting in practitioner frustrations (Table 4). The AB government/provider negotiations related to fees were highly contentious, with the government's initial offer of $20/visit for virtual visits met with physician outrage (Table 4). Reports of bankruptcy, furloughing staff, or complete practice shutdown from inadequate reimbursement were evident.

In contrast, some allied-health professionals appeared less concerned about monetary compensation. A co-owner of Lab Health Physio in Victoria, BC, explained, “We decided to offer it [virtual-care rehabilitation services] for free because we want to make sure our healthcare workers can still access physiotherapy care while clinics are temporarily closed.” (The News, April 2).

#### Patient economics

Articles noted that most physician virtual care services, including BC's CloudMD and TELUS Babylon, were covered under a provincial health plan (Table 4). Private health insurance plans, however, varied in coverage and potentially had greater financial impact on patients. Some private plans excluded newly diagnosed conditions and others required upfront payment and later reimbursement. The voices of insurance companies and providers, but not patients, appeared in insurance coverage reports.

### Virtual care model

Virtual care as a care model was addressed in nearly half (46%) of the articles (Table 5). It was positioned in relation to in-person care as both a care alternative and as a replacement (i.e., no patient choice) and particularly in projections of postpandemic virtual care. Several features of the virtual care model included accessibility, time, data gathering, technology needs, and continuity of care. Only five articles referred to the quality of virtual care delivery but did not elaborate on what this looked like or how it was achieved.

**Table 5.** Virtual Care Model Theme and Supporting Quotes

Theme	Quotes
Virtual versus in-person care	*Manitoba chief nursing officer*, “the move to virtual care isn't intended to replace in-person care, and those who need urgent attention are urged to seek it” (The Winnipeg Sun, April 28)
	*Calgary-based clinic CEO*, “virtual care has been able to come in and replace a large majority of in-person, face-to-face medical appointments” (Calgary Herald, April 4)
Improved access to care	*Calgary clinic INLIV*, “(We) offer telemedicine [through a company called Wello] to those who don't have easy physical access to health care – such as Canadians who live or work in remote rural conditions, university students studying far from home and truck drivers on the road.” (The Calgary Sun, April 4)
	*Manitoba clinic executive director*, “The benefits to mobility-restricted patients or (their) middle-aged children who would normally need to take time off to bring an elderly parent to the doctor's office are pretty obvious.” (Winnipeg Free Press, March 29).
Time	*Patient, “*There's no travel…There's more time to discuss with (the doctor) and my wife can sit comfortably on the couch taking detailed notes.” (The Spectator, April 6)
Gathering data	*ON psychiatrist* (re: video), “allows her to see people in their own environment and gives … insight into how they're functioning at home.” (The Globe and Mail, March 21)
	*BC physician* (re: blue-tooth-based stethoscopes/otoscopes), “you can listen to the heart and lungs, or see an ear drum in high definition” (The Vancouver Sun, February 27)
	*Physician,* “I do not see myself initiating video conferencing because of the strict criteria for ensuring patient confidentiality by the CPSA (College of Physicians and Surgeons Alberta).” (The Medicine Hat News, March 25)
	*Reporter*, “Its [company] terms and conditions state: ‘We may share your personal data with members of our corporate group and our partners’ and ‘personal data may be accessible by foreign government agencies under applicable law.’ The app records your consultations with doctors. This means that patient data will likely cross provincial and even national borders, where Alberta (AB) and federal privacy laws may not apply.” (Edmonton Journal, March 26)
Technology needs	*Ontario physician*, “(most of my patients) are in their 70s and 80s. Many don't have smartphones, email addresses or computers.” (Waterloo Region Record, March 12).
Continuity of care	*Alberta physician*, “physicians from my clinic have called Telus and were told they cannot see their own patients through this platform… We want to be able to see our own patients in our own critically underserved rural community where we know our local resources. (The South Peace News, March 25)
	*Ottawa physician*, “as visits become less frequent and more impersonal with the use of ‘virtual care’ there is a greater risk of worsening of chronic conditions… Some patients may ‘fall through the cracks’.” (The Winnipeg Sun, March 30)
	*Ontario physician*, “Many people rarely see their family doctors, saying they are “just too far away.” Consequently, they receive fragmented care… Instead of “losing touch” with patients, virtual care offers physicians a chance to reclaim the continuity of care that leads to really “knowing one's patients.” (The Globe and Mail, August 8)

#### Accessibility

In multiple articles, clinicians, administrators, and virtual care entrepreneurs described accessibility as an important virtual care feature. Accessibility was emphasized for patients living in remote communities and for patients with disabilities (Table 5).

#### Time

Virtual care was viewed as both time-saving (Table 5) and time-consuming. For example, patients in ON and the Yukon Territory spoke about inadequacies of virtual care hotlines—long wait times for callbacks, lack of staffing, and inability to reach anyone. Fewer articles reported patient complaints about hotlines in later months. Other articles spoke to the decreased travel time and increased flexibility afforded by virtual care.

#### Gathering data

Data gathering was a feature raised in the articles that was presented as having both value and limitations. Added value was mentioned with respect to insight into a patient's home environment and virtual examination features (e.g., wound images, vital signs) that could be gathered virtually (Table 5).

Concerns over privacy and confidentiality in data gathering were unique to articles from AB and specific to their virtual care Babylon app that had not undergone a privacy assessment before launch (Table 5). In June, news articles reported that Alberta's privacy commissioner had launched an investigation into the app's “compliance concerns” and adherence to the Personal Information Protection Act (Edmonton Journal, June 11). At the time of writing, results from this investigation had not been announced. Researchers and clinicians raised concerns about the disconnect between strict needs for patient privacy and virtual care companies' liberal data-sharing policies.

#### Technology needs

Lack of access to technology and connectivity to support video-based virtual care was noted in many articles (Table 5). News stories implied that virtual care was an easier way to access health care using a smartphone, tablet, or computer connected to the internet. However, this was countered with reports of lack of stable internet connection, technical issues, and inundation of phone lines, notably in BC, AB, ON, and the Yukon.

#### Continuity of care

Many articles discussed lack of care continuity experienced with virtual care apps when practitioners are unable to see their own patients, patient consultation results are not shared with the patient's provider, and physicians working through virtual care apps who lack access to patients' medical records (Table 5). In addition, there was concern that less frequent or more impersonal virtual care might worsen health outcomes. Conversely, improvements in care continuity could result from virtual care of patients in remote areas (Table 5).

#### Future of virtual care

Predictions for postpandemic use of virtual care were considered in several articles. An overwhelming volume of articles (*n* = 38) from March through May discussed integration and use of virtual care as the “new normal.” The medical director of a Calgary-based private clinic said, “telemedicine and offering virtual care is here to stay… we need to embrace the change” (Calgary Sun, April 4).

From June through August (*n* = 25), the discussion shifted. Whereas early articles spoke to virtual care transitioning, later articles discussed allowing more in-person visits, as restrictions were lifted, and highlighted that virtual care could not replace in-person care. One family doctor said, “I don't believe it's going to replace the traditional way of providing health care, but at the same time I think it is something we should integrate long-term” (Observer, June 26).

Many acknowledged that virtual care would remain at the forefront of patient care and that benefits in time, cost, convenience, and decreased PPE usage and exposure risks could not be overlooked. Providers noted that patient care would likely become a hybrid of virtual and in-person care. Patient voices were again absent from this reporting.

## Discussion

In Canada, as elsewhere, the COVID-19 pandemic served as a catalyst to increase virtual care uptake,^13^ with virtual care quickly becoming the dominant outpatient-care delivery model. However, this news media analysis revealed that the initial push to implement virtual care quickly tapered off after only 3 months. Whether this reflects a normalizing of virtual care, a slow return to in-person care, or both, the loss of news media attention may signal a loss of momentum in advancing virtual care as a sustainable health care delivery option. For example, funding models, mainstream integration, and quality standards are all continuing areas needed for sustainable virtual care and it is not clear that government funding for virtual and telephone care will continue postpandemic.

While many aspects of virtual care during the first 7 months of COVID-19 were framed positively in the news media, negative views about confidentiality and funding may make virtual care less attractive. Media pressure, however, has the potential to push government action to invest in infrastructure to improve virtual care provision.^14^ This endeavor requires a vision for creating a *better normal*, not simply returning to normal once the pandemic resolves.

Our findings also uncovered a lack of patient voice and choice regarding virtual care in news article coverage. To create sustainable change, it is imperative to understand how virtual care is perceived and experienced by patients and nonphysician providers. In a large Canadian (*n* = 58,000) Infoway survey, the vast majority (91%) of Canadians who received virtual care during the pandemic were satisfied with the experience, 86% found virtual care tools important alternatives to in-person visits, 76% were willing to use virtual care after the pandemic, and nearly 6 in 10 Canadians wanted more information about digital health care services.^15^ News media could serve as an important source of this information and for capturing patient opinions.

### Safety

Safety was a main driver for virtual care implementation. Virtual and in-office care provided by primary care clinicians is vital during disasters and pandemics to route people away from ED and hospital use while providing continuity of care and urgent, nonemergency care.^5,13,14^

News media provision of concrete behavioral recommendations, as with instructional messaging, during pandemic times can increase perceived self-efficacy and improve behavioral intentions.^16^ However, we found that safety and protection messages were often contradictory, which may undermine trust, an essential element of risk communication.^17^ Furthermore, reassuring and instructional messaging need to be better balanced to avoid delayed health care; reassuring messages were mostly absent after May 2020 with potentially dire consequences.^18,19^

It is likely difficult for readers to distinguish between recommendations made by credible authorities and opinions by individuals, including individual providers; news media should be encouraged to clearly identify the most trusted sources. Coordinated communication at the national, provincial, and local levels is a critical component of pandemic readiness,^3^ and researchers should explore the role that news media plays in patients' care seeking decisions and potential harms that occur with unsubstantiated claims.

### Limitations

Despite our pan-Canadian news media coverage of virtual care during COVID-19, results were limited to English language, excluding French-speaking regions of Canada. In addition, Canadian Newsstream is a fairly exhaustive database for newspaper articles but does not contain articles from online news outlets. Our analyses, however, provide a broad snapshot of reports of virtual care during the first 7 months of Canada's response to COVID-19. While these reports may not fully reflect the realities of how virtual care has been or will be integrated into ongoing care models, they can serve to change public perceptions that can lead to the adoption or exclusion of these alternatives to usual care.

### Future directions

While our study clearly identified the pandemic's role as a virtual care catalyst largely for patient, provider, and health care system protection and the critical role of government financing, gaining public/patient perspectives on virtual care that were largely missing from the news media is vital for gauging the overall receptivity and long-term sustainability.

Policy makers would also do well to work with the media to craft messages that balance information with reassurance. Further research into the influence of virtual care media messaging on public response, attitudes, and satisfaction with virtual care would be informative as would understanding any differences in public response to messaging between the local and national papers. Zhang and Chen present a Government–Expert–Public Risk Communication model that might be helpful in this regard.^17^

Finally, the views about virtual care as a care model point to several areas for exploration including cost-efficient ways to expand internet access while protecting patient confidentiality, and provider needs for training in virtual care platforms and development of team workflows that optimize virtual care integration. These in turn have the potential for directing a research agenda and public policy around adoption of virtual care.

## Conclusion

Canadian news articles in the first 7 months of the COVID-19 pandemic portrayed the pandemic as a catalyst for widespread virtual care uptake, in large part, to protect patients, providers, and the health care system. Messages to the public were instructional and reassuring, but sometimes contradictory, which may have undermined trust and caused patients to avoid seeking care. When provincial funding for virtual care was delayed, economic discussions were contentious. Given the clear benefits of virtual care, adequate funding and solutions to technology needs are vital for making virtual care sustainable.

## Abbreviations Used

AB: Alberta
BC: British Columbia
COVID-19: coronavirus disease
CPSA: College of Physicians and Surgeons Alberta
DaSHI: Data Science and Health Informatics
ED: emergency department
HBT: Health Benefits Trust
NB: New Brunswick
NS: Nova Scotia
ON: Ontario
PBC: Pacific Blue Cross
PPE: personal protective equipment
QC: Quebec
SK: Saskatchewan

## Notes

^*^
Overlap of themes occurred in some articles and resulted in some categories and subcategories adding to more than 100%.
